# Serum CEACAM1 Level Is Associated with Diagnosis and Prognosis in Patients with Osteosarcoma

**DOI:** 10.1371/journal.pone.0153601

**Published:** 2016-04-13

**Authors:** Haiying Yu, Jian Yu, Yanjun Ren, Yun Yang, Xing Xiao

**Affiliations:** 1 Department of Radiology, Shandong Cancer Hospital and Institute, Affiliated to Shandong Academy of Medical Science, Jinan, Shandong, China; 2 Department of Orthopedics, Caoxian People’s hospital, Shandong, China; 3 Department of Orthopedics, Shandong Provincial Qianfoshan Hospital, Jinan, Shandong, China; Faculté de médecine de Nantes, FRANCE

## Abstract

Carcinoembryonic antigen related cell adhesion molecule 1 (CEACAM1) is a trans-membrane multifunctional cell adhesion molecule associated with tumor cell proliferation, apoptosis, angiogenesis, invasion, and migration during tumor development. In the present study, we evaluated serum CEACAM1 level in osteosarcoma patients to explore its diagnostic and prognostic value for this particular malignancy. Sera from 113 patients with primary osteosarcoma, 98 patients with benign bone tumors and 126 healthy controls were obtained. Serum CEACAM1 level was measured with ELISA and correlation with clinicopathological characteristics was further analyzed. Receiver operating curves (ROC), Kaplan-Meier curves, and log-rank analyses as well as Cox proportional hazard models were used to evaluate diagnostic and prognostic significance. The results revealed that serum CEACAM1 level was significantly higher in osteosarcoma patients compared to benign bone tumors and healthy controls (455.2 ± 179.9 vs 287.4 ± 103.2, 260.8 ± 109.7 pg/ml, respectively). Osteosarcoma patients with larger tumors, later-tumor stages, low tumor grades, and distant metastases had much higher CEACAM1 compared to those with smaller tumors, earlier tumor stages, high tumor grades and non-distant metastases (P < 0.05 for all). Multivariate logistic regression analysis confirmed that high serum CEACAM1 level was an independent risk factor for distant metastases (OR = 3.02, 95%CI 1.65–4.17). To distinguish osteosarcoma patients from those with benign bone tumor and healthy controls, ROC/AUC analysis indicated an AUC of 0.81 (sensitivity 0.61; specificity 0.89) and an AUC of 0.77 (sensitivity 0.57; specificity 0.92), respectively. Osteosarcoma patients with higher CEACAM1 had relatively lower survival compared to those with low CEACAM1 (P < 0.01), and multivariate analyses for overall survival revealed that high serum CEACAM1 level was an independent prognostic factor for osteosarcoma (HR = 1.56, 95%CI 1.23–3.28). The present study suggested that elevated serum CEACAM1 level might be a novel diagnostic and prognostic biomarker for osteosarcoma patients.

## Introduction

Osteosarcoma (OS) is the most common primary malignant bone cancer and it manifests mainly in children, adolescent and young adults, comprising approximately 20% of all bone tumors and 5% of pediatric tumors overall[1–3]. Osteosarcoma tends to occur in long bones metaphysis and is most frequently found in the distal femur, proximal tibia, and humerus. Osteosarcoma is characterized by a local invasion of bone and soft tissues, loss of affected extremity functions and distant metastases. Typically, this cancer presents as pain and swelling in the affected bone and this is initially mild but becomes highly aggressive. Approximately 15–20% of OS patients will have clinically detectable metastases at presentation, and more than 85% of metastases occur in the lung [4]. For OS patients lacking metastases at diagnosis, the combination of aggressive surgical resection, chemotherapy and radiotherapy offer significant improvements for five-year survival [3]. However, most OS patients are diagnosed at advanced stages and long survival after diagnosis of advanced OS is rare [5,6]. Thus, we need better molecular biomarkers to detect OS earlier and better prognostic indicators to assist with treatment and improve patient survival.

Carcinoembryonic antigen related cell adhesion molecule 1 (CEACAM1) is a trans-membrane multifunctional cell adhesion molecule of the CEACAM family, which is a member of the immunoglobulin superfamily[7]. CEACAM1 is broadly expressed in many epithelial, endothelial, and hematopoietic cells such as monocytes and natural killer cells[8]. Researches indicate that CEACAM1 has many biological functions and can regulate immune responses, neovascularization and insulin clearance [9]. During tumor development, CEACAM1 is associated with tumor cell proliferation, apoptosis, angiogenesis, invasion and migration [10]. Due to its trans-membrane glycoprotein property, CEACAM1 can exist in trans-membrane form as well as in soluble form, as found in urine and saliva [11,12]. Until now, several reports have focused on the role of soluble CEACAM1 in tumor patients and found elevated serum CEACAM1 level was relevant to tumor presence, progression and survival for patients with non-small cell lung, pancreatic cancers and malignant melanoma [13–15]. These data support a potential role for serum CEACAM1 as a novel molecule biomarker for tumor patients, but the relevance of serum CEACAM1 in OS is unclear. Thus, we studied this aspect of OS to clarify the significance of serum CEACAM1 in patients with OS and evaluated its potential diagnostic and prognostic value.

We measured soluble CEACAM1 using ELISA and assessed its relationship with tumor characteristics as well as its potential as a novel non-invasive diagnostic and prognostic indicator of OS. Further, we measured trans-membrane CEACAM1 expression in tumor tissues using real-time PCR (RT-PCR) and Western blotting.

## Materials and Methods

### Participants

Patients (N = 113) with primary OS treated in the department of orthopedics of Shandong Qianfoshan Hospital (Jinan, China) between July 2008 and December 2012 were enrolled. OS diagnoses were based on clinical and histological examination of resected specimens from primary OS. All patients had no history of other cancers and underwent no other prior treatment including chemotherapy or radiotherapy when first diagnosed with primary osteosarcoma. Of these, 98 patients had benign bone tumors including osteochondromas, chondromas, bone cysts, and ossifying fibromas were included. All benign bone tumors were also diagnosed based on histology. Age- and gender-matched 126 controls, unrelated to OS patients, were recruited and had no orthopedic disease or cancer. This study as well as follow-up study were approved by the Ethics Committee of Shandong Qianfoshan Hospital (Ethical Approval Number: QFSYY200801004, Jinan, China) and the written informed consent was obtained from all participants.

### Data collection and follow-up

Patient demographic and clincopathology data (Enneking staging system, ESS), tumor location, metastasis and pathological type were collected from medical records at the time of diagnosis. OS patients received standard therapeutic procedures including neoadjuvant chemotherapy and surgical resection with a wide or radical margin followed by adjuvant chemotherapy. Chemotherapeutic response was classified as poor (<90% tumor necrosis) or good (>90% tumor necrosis) via histology of tumor specimens [16]. After surgery, all OS patients were recruited for follow-up monitoring. Disease-free survival time was measured as the time from the surgery day until the date of cancer reoccurrence or patient death or the day of the last live follow-up to represent disease progression. The latest follow-up day was July 28, 2015, and median follow-up for overall survival was 32.6 months (range: 3–46 months).

### ELISA for soluble CEACAM1 in sera

Venous blood samples (~5 ml) were obtained from each participant when first diagnosed with OS by authors (J.Y and Y.R). Blood was allowed to coagulate in a serum separator tube. Samples were centrifuged (3 000 x g) for 10 mins and serum was transferred to tubes and stored at -80°C until ELISA quantification of soluble CEACAM1. Serum CEACAM1 level was measured with a commercial ELISA kit (RayBiotch, GA, USA) according to the manufacturer’s instructions[17]. Briefly, a 96-well plate was pre-coated with anti-human CEACAM1 antibodies and sera samples as well as recombinant human CEACAM1 were diluted with sample dilution buffer and added into wells. Plates were incubated at room temperature for approximately 2 h. After CEACAM1 was bound to the wells by the immobilized antibody, wells were washed four times with a washing buffer. Then biotinylated anti-human CEACAM1 antibody was added into wells, and incubated at room temperature for 1 h. After washing four times, HRP-conjugated streptavidin was added after removing unbound biotinylated antibody. Wells were washed once more and TMB substrate solution was added as well as stop solution. At last, optical density was measured at 450 nm using a micro-plate reader (Kehua, Shanghai, China). According to the standard curve generated by serial dilution of the recombinant human CEACAM1, serum soluble CEACAM1 from each individual was calculated.

### Real-Time Quantitative PCR (RT-PCR)

Surgically dissected OS tissue specimens and corresponding noncancerous bone tissues were collected and stored at -80°C. Total RNA was extracted from frozen tissues using TRIzol (Invitrogen, CA, USA), following the manufacturer’s instructions. Then, ~1 μg of total RNA from each sample was used for cDNA synthesis using a reverse transcription kit (Takara, Japan). After obtaining cDNA, quantitative real-time PCR was performed (20 μl final volume), with qRT-PCR conditions as follows: 98°C for 10 s, 60°C for 15 s, 72°C for 30 s, for 40 cycles. CEACAM1 mRNA expression was estimated relative to GAPDH using the equation 2^-ΔΔCt^(ΔCt = Ct_CEACAM1_-Ct_GAPDH_). Primers used were as follows: 5′-AATGTTGCAGAGGGGAAGGA-3′ (forward) and 5′-tctgggtgacgttctggatc-3′ (reverse) for CEACAM1. And 5′-ccagaacatcatccctgcct-3′ (forward) and 5′-cctgcttcaccaccttcttg -3′ (reverse) for GAPDH.

### Western blotting

Frozen tumor tissues and adjacent tissues were homogenized on ice with a glass homogenizer and lysed in RIPA buffer containing protease inhibitor (cOmplete, Sigma, CA, USA) and PMSF. Lysates were sonicated and centrifuged at 13,000 rpm at 4°C for 5 min. Then samples were precipitated, and supernatants were collected. A BCA assay kit (Beoytime Biotech, Shanghai, China) was used to measure protein concentration and extracted proteins were subjected to 8% SDS-PAGE, then separated proteins were transferred onto PVDF membranes (Millipore, MA, USA). The PVDF membrane was blocked with TBST (TBS buffer with 0.1% Tween-20) containing 5% skim milk and incubated with human CEACAM1 antibody (1ug/ml; Clone 283324; Monoclonal Mouse IgG1, R&D System, USA) and GAPDH antibody (1:1000 dilution, sc-32233; Mouse Monoclonal IgG1, Santa Cruz, USA) at room temperature for 2 h. Subsequently, samples were incubated with HRP-conjugated secondary antibody (1:5000 Dilution, Beoytime Biotech, Shanghai, China). Signals were captured using an HRP Chemiluminescent kit (Beoytime Biotech, Shanghai, China) and CCD camera image system (Bio-Rad, CA, USA).

### Statistical Analysis

One-way ANOVA was applied to determine differences in serum CEACAM1 level among OS, benign bone tumor and control samples. A paired Student’s t-test was used to assess CEACAM1 mRNA expression between tumor tissues and tumor-adjacent tissues. Nonparametric received operating characteristic (ROC) curves were generated to assess diagnostic efficiency. Survival probabilities were estimated with a Kaplan–Meier analysis, and significant differences were analyzed with a log-rank test. Multivariate analysis of prognostic factors was performed using a COX regression analysis. All analyses were conducted with SPSS 18.0 (SPSS Inc., Chicago, IL). p < 0.05 was considered to be statistically significant.

## Result

### Patient characteristics and serum CEACAM1 level

**Fig 1:**
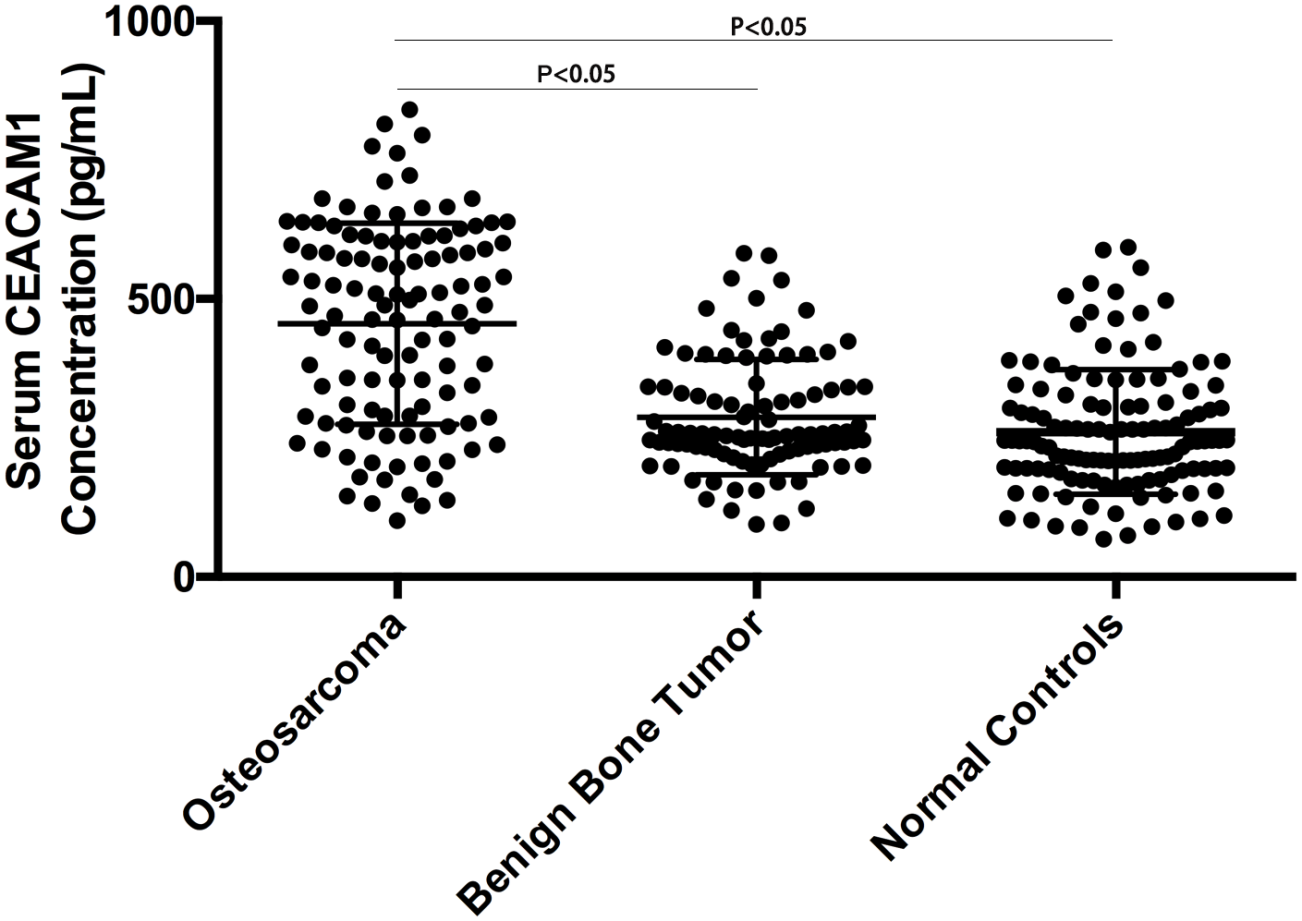
Serum CEACAM1 concentration among OS patients, benign bone tumor and healthy controls.

Patient characteristics are noted in Table 1. There were no significant differences among patient characteristics. Serum CEACAM1 level was elevated in OS patients compared to those with benign bone tumors and controls (455.2 ± 179.9 vs 287.4 ± 103.2, 260.8 ± 109.7 pg/ml, respectively; Fig 1). Patients with benign bone tumors had slightly higher serum CEACAM1 level than healthy controls, but this was not significant (P = 0.17).

**Table 1:** OS patient characteristics.

Characteristic	Osteosarcoma (n = 113)	Benign tumor (n = 98)	Healthy controls (n = 126)
Age (Years)			
≤20	67	67	85
>20	46	31	41
Gender (n, %)			
Male	70	62	84
Female	43	36	42
Tumor Location			
Femur	69		
Tibia	29		
Other	15		
Tumor Size (cm)			
<6	71		
≥6	42		
Tumor grade			
Low	52		
High	61		
Clinical Stage			
IIA	33		
IIB	60		
III	20		
Distant Metastasis			
Negative	80		
Positive	33		
Response to chemotherapy			
Poor	68		
Good	45		

### Association between serum CEACAM1 level and clinicopathological features in osteosarcoma patients

**Fig 2:**
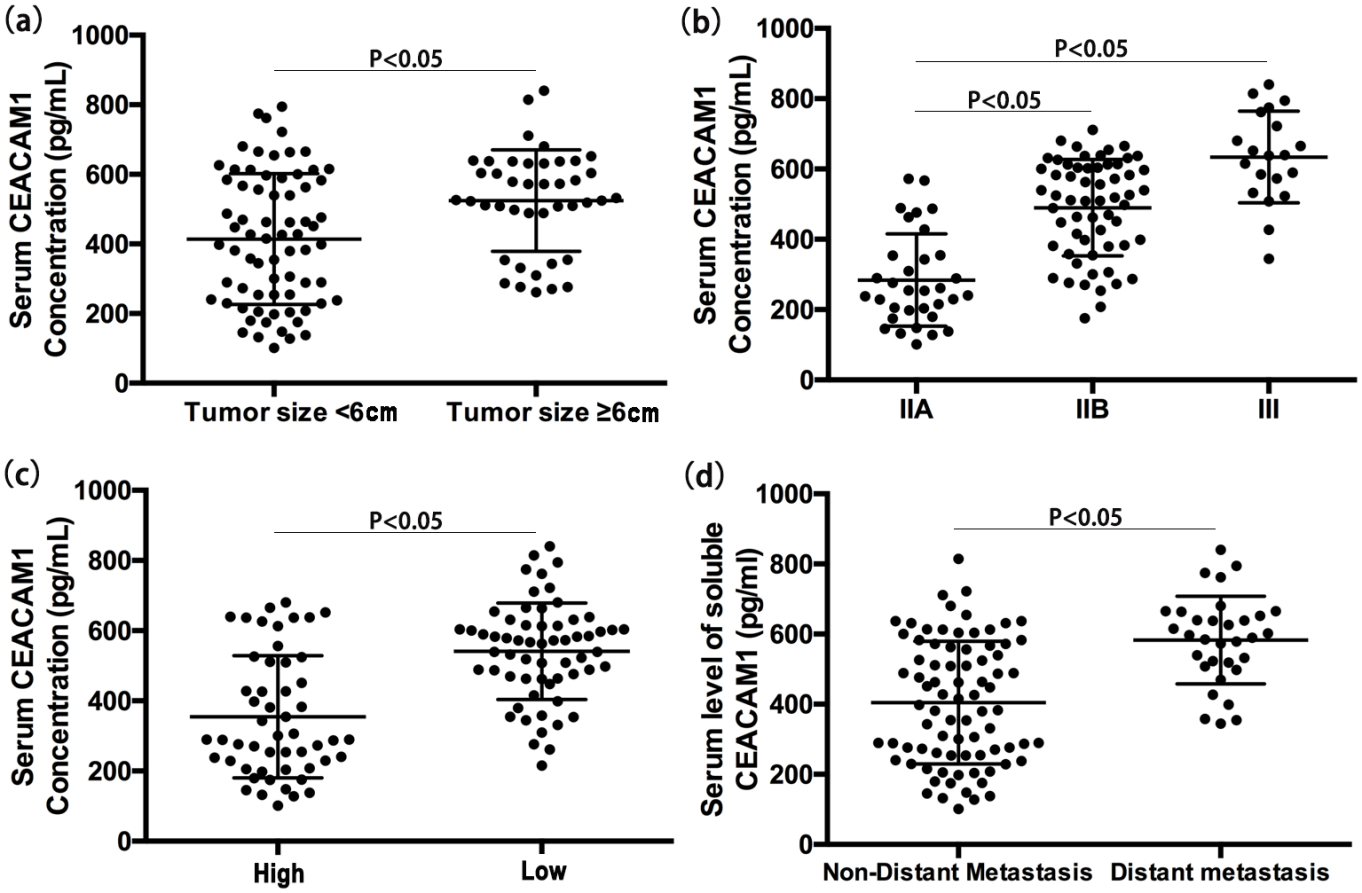
Correlation analysis between serum CEACAM1 level and OS patients characteristics; Serum CEACAM1 level among OS patients with different tumor size (a); different clinical stages (b); different tumor grades (c); with and without distant metastases (d).

Serum CEACAM1 level was not correlated with age or gender (P = 0.22, P = 0.46; respectively) or with chemotherapeutic response (P = 0.69). OS patients with femur tumors had greater serum CEACAM1 level than OS patients with tumors at other sites, but this was not significantly different (P = 0.08, P = 0.17; respectively; S1 Fig). Tumors greater than 6 cm were associated with higher serum CEACAM1 level compared to tumors smaller than 6 cm (P < 0.05, Fig 2a). Serum CEACAM1 level varied among patients with different clinical stages, and stage III patients had significantly higher CEACAM1 than those with IIA or IIB stages (P < 0.05 for both). Similarly, IIB stage patients had higher CEACAM1 than those with IIA stage cancers (P < 0.05, Fig 2b). Serum CEACAM1 level was higher in OS patients with low tumor grades (P < 0.05, Fig 2c) and OS patients with distant metastases (P < 0.05, Fig 2d).

### Correlation analysis of serum CEACAM1 level with distant metastases, advanced clinical stage

Using multivariate logistic regression analysis, we noted that high serum CEACAM1 level was an independent risk factor for distant metastases for OS patients after adjusting for age and gender (OR = 3.02, 95%CI 1.65–4.17). Higher serum CEACAM1 level was found in OS patients with advanced clinical stages but increased CEACAM1 was not found to be an independent risk factor for late clinical stages (IIB/III) (OR = 1.65, 95%CI 0.62–2.55).

### ROC analysis of serum CEACAM1 level in osteosarcoma patients

**Fig 3:**
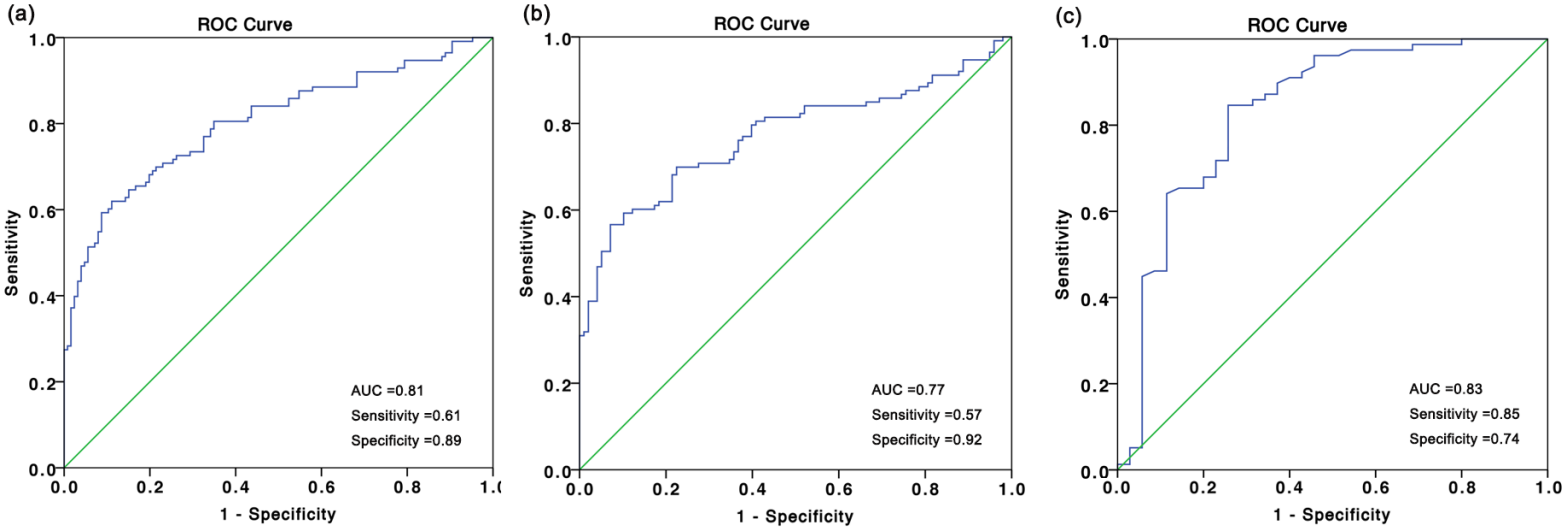
ROC analysis for serum CEACAM1 for distinguishing OS patients from benign bone tumors or healthy controls; ROC for serum CEACAM1 differentiating OS patients from controls (a); from those with benign bone tumors (b); distinguishing OS patients with IIB/III stages from those with IIA stages (c).

ROC/AUC analysis revealed sensitivity and specificity of different serum CEACAM1 concentrations. ROC curve analysis illustrated that serum CEACAM1 level was a potential biomarker for screening OS patients form controls (AUC of 0.81; sensitivity 0.61; specificity 0.89, Fig 3a). Serum CEACAM1 could also distinguish OS patients from those with benign bone tumors (AUC of 0.77; sensitivity 0.57; specificity 0.92, Fig 3b). To assess the potential of CEACAM1 as a diagnostic biomarker to distinguish advanced cancer patients (IIB/III) from early clinical stages (IIA), we got an AUC of 0.83 (95%CI 0.74–0.92) with a sensitivity of 0.85 and a specificity of 0.74 (Fig 3c).

### Correlation analysis of serum CEACAM1 level and prognosis in osteosarcoma patients

**Fig 4:**
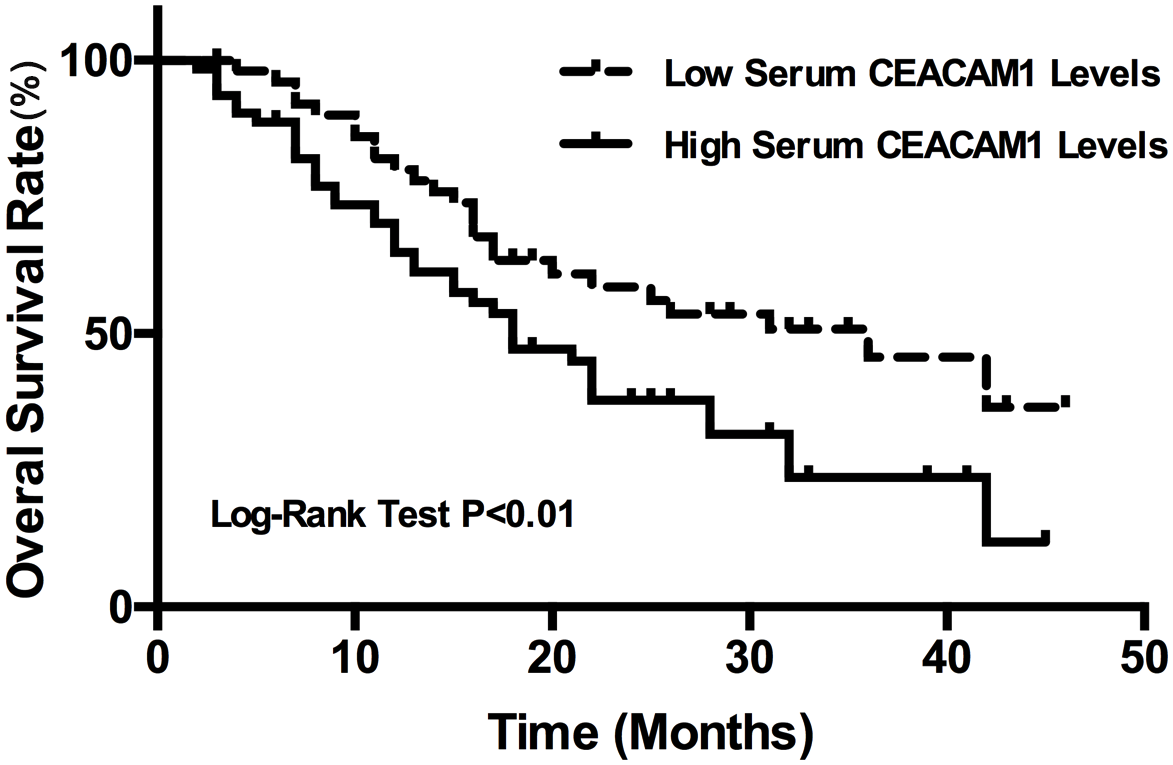
Kaplan-Meier survival curves; overall survival for OS patients with high serum CEACAM1 level compared to those with low CEACAM1 (P < 0.01).

The enrolled 113 OS patients received standard therapy and all completed the follow-up. Overall survival was defined as the time to date of tumor reoccurrence or death or the last follow-up. Stratifying OS patients by average serum CEACAM1 values (455.2 pg/ml), we observed high and low groups. According to Kaplan-Meier and log-rank tests, low CEACAM1 group had greater survival compared to those with high CEACAM1 (P < 0.01, Fig 4). To find whether CEACAM1 could be a useful prognostic assessment factor for OS, Cox univariate and multivariate regression analysis revealed that high CEACAM1 was an independent prognostic molecule biomarker for poor prognosis (HR = 1.56, 95%CI 1.23–3.28; Table 2).

**Table 2:** Univariate and multivariate Cox proportional hazard analysis for OS patient overall survival.

Factor	Category	Univariate		Multivariate	
		HR (95% CI)	P	HR (95% CI)	P
Age	≥20/<20	0.68 (0.38–1.67)	0.63		
Gender	Male/Female	1.55 (0.76–3.56)	0.37		
Tumor location	Femur + Tibia/other	1.08 (0.66–1.87)	0.34		
Tumor size	≥6/<6 cm	1.93 (0.93–2.78)	0.28		
Tumor grade	Low/High	2.64 (1.76–4.02)	<0.01	2.18 (0.76–4.65)	0.26
Clinical stage	IIB+III/ IIA	4.38 (2.12–6.53)	<0.01	2.05 (1.57–4.30)	<0.01
Distant metastases	Yes/No	5.61(3.28–9.45)	<0.01	3.81 (2.35–6.09)	<0.01
Chemotherapy response	Poor/Good	4.88(2.63–7.03)	<0.01	2.64 (1.59–4.36)	<0.05
Serum CEACAM1	High/Low	2.78 (1.78–4.21)	<0.01	1.56 (1.23–3.28)	<0.05

### Production of CEACAM1 in osteosarcoma tumor tissue

**Fig 5:**
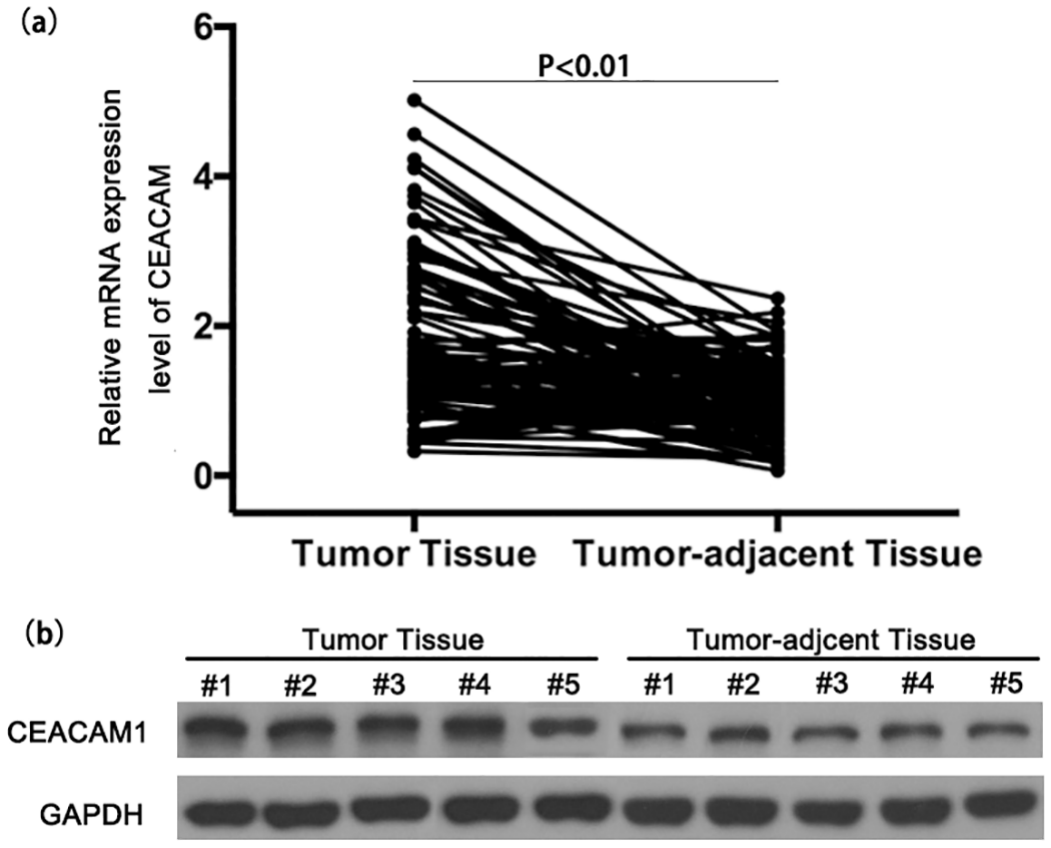
CEACAM1 expression level in OS tumor tissue; CEACAM1 mRNA (a) and protein (b) expression of tumor tissues and tumor-adjacent tissues.

RT-PCR analysis revealed that mRNA expression levels of CEACAM1 were significantly increased in OS tumor tissues compared to tumor-adjacent tissue (Fig 5a). In compliance with mRNA expression, western blotting analysis also showed that the proteins levels were increased in OS tumor tissues (Fig 5b and S2 Fig). These data may suggest increased production of CEACAM1 was the potential source of serum CEACAM1.

## Discussion

OS is an aggressive malignancy associated with poor prognosis and most OS patients are diagnosed at advanced stage which worsens chances for survival. Lack of efficient early diagnostic tumor biomarkers to detect metastases and predict survival is a challenge worthy of study. Noninvasive serological biomarkers can allow OS patients to undergo surgery and allow better chemotherapy or radiotherapy planning during early malignancy, and some potential biomarkers have been reported in the previous literatures [18–23]. We assessed diagnostic and prognostic value of CEACAM1 as a non-invasive serological biomarker and we found that serum and tissue CEACAM1 were significantly elevated in OS patients and elevated serum CEACAM1 level was correlated with lower tumor grade, more advanced clinical stage and distant metastases. Serum CEACAM1 may be a diagnostic biomarker to distinguish OS from benign bone tumors and controls, and can distinguish advanced from early OS stages. Also, elevated serum CEACAM1 level was an independent prognostic biomarker for these patients.

CEACAM1 is important to tumor development and altered CEACAM1 expression has been reported in many cancers [7,10,24]. However, CEACAM1 expression correlative studies for cancer patients are controversial. Overexpression of CEACAM1 is widely reported, but some studies report that decreased expression occurs in some tumor types and at different tumor stages [24]. No report has been published to connect CEACAM1 expression to aspects of OS. So, we assessed CEACAM1 expression in OS and noted that CEACAM1 mRNA and protein were significantly increased in OS tissue compared to tumor-adjacent tissue. The diagnostic and prognostic values of serum soluble CEACAM1 in cancer patients is receiving scrutiny; CEACAM1 as a diagnostic tumor marker has been reported for several different cancer types. Simeone’s group reported the potential of CEACAM1 as a serum tumor biomarker in pancreatic carcinoma and noted significantly elevated CEACAM1 had comparable specificity and sensitivity to CA19-9, a current standard serum biomarker for pancreatic cancer [13]. Zhou’s group reported that CEACAM1 expression was significantly increased in early stage non-small-cell lung cancer (NSCLC) patients indicating that CEACAM1 could be used for early diagnosis [15]. In addition, elevated serum CEACAM1 level may be a useful indicator to identify breast cancer[17]. Thus, CEACAM1 has the potential to be a novel tumor biomarker and warrants further study.

We are the first to report the utility of CEACAM1 as a serum diagnostic biomarker for OS and soluble serum CEACAM1 from OS patients was found to discriminate cancer from non-cancerous lesions. This finding was consistent with previous studies to show that serum CEACAM1 level was correlated with the presence of cancer. According to the literatures, the diagnostic efficiency of serum CEACAM1 varied among cancers, but as a single diagnostic biomarker, high AUCs for serum CEACAM1 were noted in pancreatic cancer and NSCLC patients (AUCs of 0.936 and 0.96, respectively) [13,15]. For os, we noted that AUCs were relatively lower for cancer (0.81), which is comparable to breast cancer values[17]. Also, ROC data confirmed low sensitivity for CEACAM1 to differentiate OS patients from those with benign bone tumors and controls. Other serum biomarkers had low sensitivity as well and increasing this facet of biomarkers remains a challenge[25]. Previous reports have shown that simultaneous detection of multiple biomarkers may improve the sensitivity without compromising specificity. Recently, the development protein microarrays and immunobead assays have improved the simultaneous detection of serological biomarkers and this could overcome low sensitivity issues [26]. Combining CEACAM1 with other biomarkers for cancer has also offered promising results [13,19]. Yang’s group found that combining serum CEACAM1 with CA15-3 increased the diagnostic accuracy of AUC to 0.940 (sensitivity 0.79; specificity 0.97) when CEACAM1 was used with CA15-3 for breast cancer. Also, the combination of CEACAM1 and CA19-9 had significantly higher diagnostic accuracy for pancreatic cancer than using either marker alone. We studied CEACAM1 alone in the present study, so combination studies are a future goal.

Many factors can modify OS prognoses, including patient demographics, tumor size and site, clinical cancer stage and chemotherapeutic response. Here, we report that serum CEACAM1 level was significantly and independently positively correlated to OS prognosis. Also, in OS patients, tumor grade and clinical stage, distant metastases and chemotherapeutic response were significantly associated with poor OS prognosis. However, no other variable such as age, gender, tumor size and tumor location was associated with poor prognosis in the present study. Thus, we thought these prognostic factors were assessed or determined by varied criteria and different background, which made it inconsistent among different studies. Distant metastases and chemotherapeutic response were the most important and accurate prognostic determinants and we noted that these were positively correlated to CEACAM1 [27,28], which was usually a late-cancer finding[29]. Also, our data agreed with Sivan who reported clinical significance of elevated serum CEACAM1 in malignant melanoma was superior to currently most used delayed-type hypersensitivity (DTH)[14].

Although the molecular basis of cancer is under investigation, how to detect and monitor OS at early stages is not well studied. Most studies focus on specific overexpression of secreted and easily measured proteins in cancer cells that are not expressed in corresponding normal cells [30]. Sera samples from all participants were collected at the time of diagnosis and all were stored at −80°C during the follow-up period. Measurement of serum CEACAM1 level was performed blindly. We used the commercial ELISA kit, which is a quantitative detection method for serum CEACAM1 analysis in the present study. These advantages rendered our results reliable and the analysis attractive in the clinical setting. Nevertheless, the present study also has several limitations. Our study had few subjects, so we should attempt this work in a large cohort to verify diagnostic efficiency of serum CEACAM1 for OS patients. Also, we used a cross-sectional design, so any association between serum CEACAM1 level and OS risk was not evaluated. Also the source of high serum soluble CEACAM1 was not clear; higher CEACAM1 was noted in OS tumor tissues, but whether higher serum CEACAM1 were totally attributable to CEACAM1 overexpression in that tumor tissue requires further exploration. In conclusion, the present study suggested serum CEACAM1 might be a diagnostic and prognostic biomarker for OS.

## Supporting Information

S1 Fig Correlation analysis of serum CEACAM1 level with tumor sites in OS patients. (TIFF)

S2 Fig Quantification of CEACAM1 expression between OS tumor tissue and tumor-adjacent tissue using densitometric analysis. (TIFF)

S1 File Raw-Data about characteristics of all participants. (XLSX)
