# Supplementary figures and images for: Serum CEACAM1 Level Is Associated with Diagnosis and Prognosis in Patients with Osteosarcoma
Source: PLoS One. 2016 Apr 13;11(4):e0153601. doi: 10.1371/journal.pone.0153601 (PMC4830595; doi:10.1371/journal.pone.0153601)

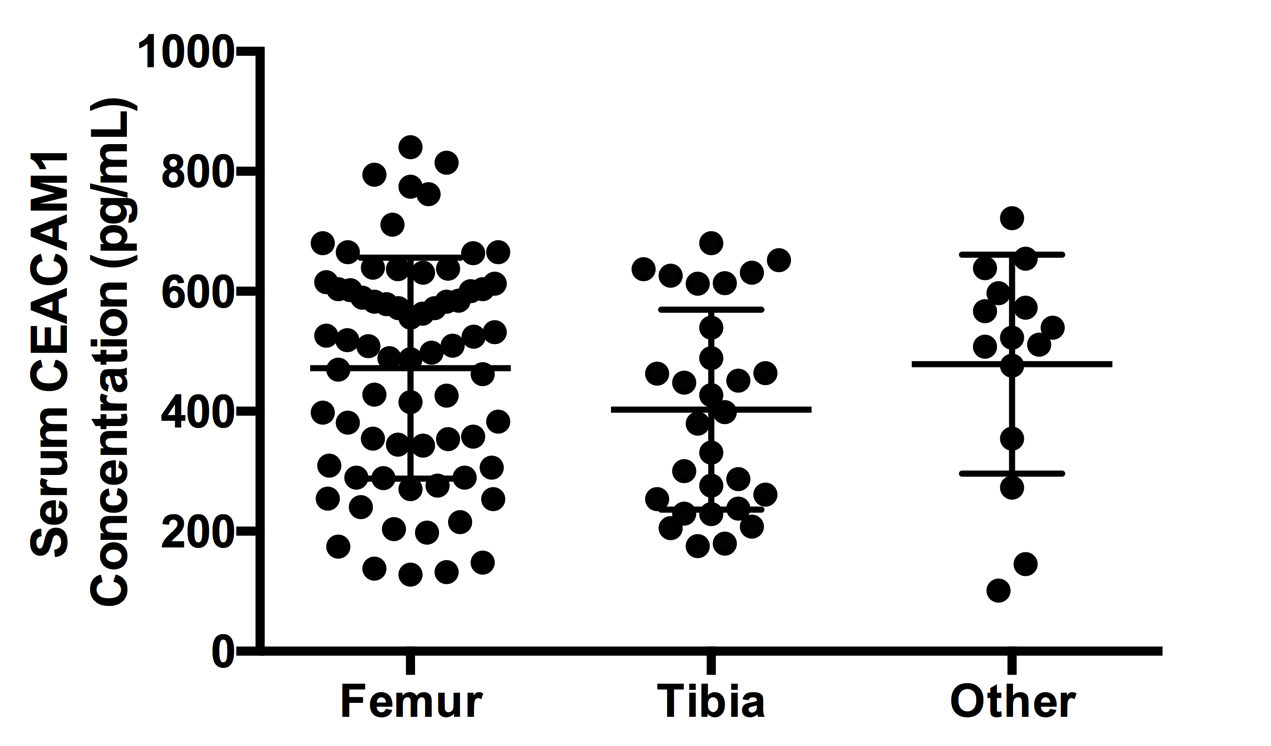

Supplement: S1 Fig — (TIFF) [file pone.0153601.s001.tiff]

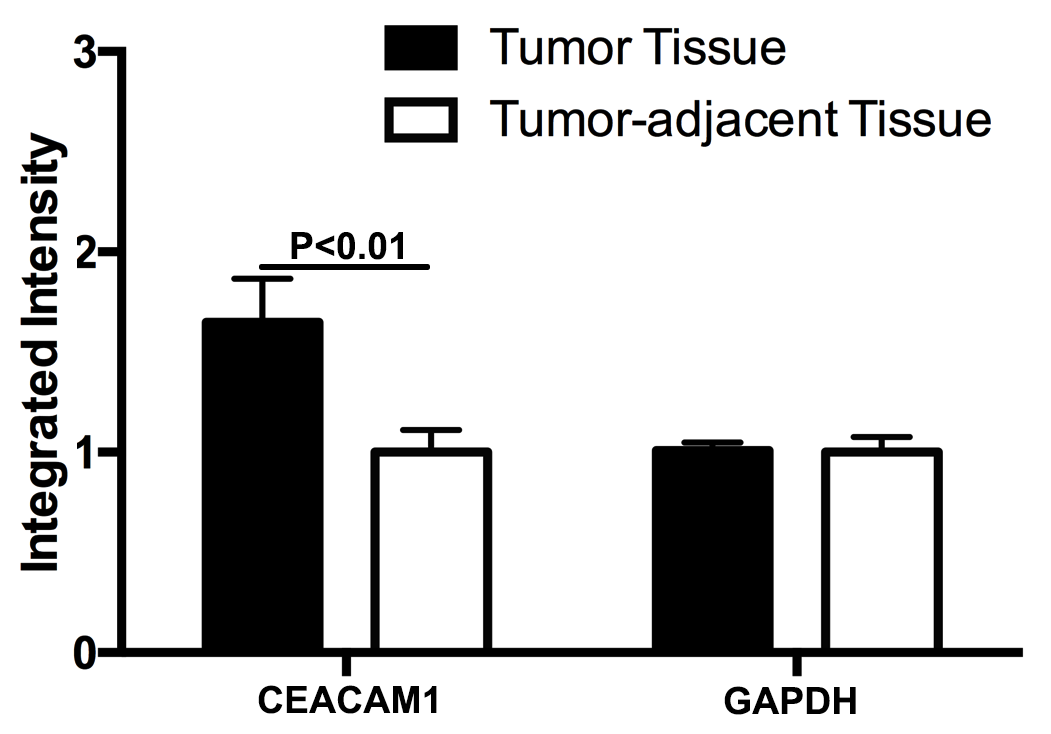

Supplement: S2 Fig — (TIFF) [file pone.0153601.s002.tiff]
